# Covalent attachment of the plant natural product naringenin to small glass and ceramic beads

**DOI:** 10.1186/1472-6769-5-3

**Published:** 2005-10-10

**Authors:** Yuhua Lu, Niloufer G Irani, Erich Grotewold

**Affiliations:** 1 Department of Plant Cellular and Molecular Biology and Plant Biotechnology Center, The Ohio State University, Columbus, OH 43210, USA

## Abstract

**Background:**

Natural products have numerous medicinal applications and play important roles in the biology of the organisms that accumulate them. Few methods are currently available for identifying proteins that bind to small molecules, therefore the discovery of cellular targets for natural products with pharmacological activity continues to pose a significant challenge in drug validation. Similarly, the identification of enzymes that participate in the biosynthesis or modification of natural products remains a formidable bottleneck for metabolic engineering. Flavonoids are one large group of natural products with a diverse number of functions in plants and in human health. The coupling of flavonoids to small ceramic and glass beads provides a first step in the development of high-throughput, solid-support base approaches to screen complex libraries to identify proteins that bind natural products.

**Results:**

The utilization of small glass and ceramic beads as solid supports for the coupling of small molecules was explored. Initial characterization of the beads indicated uniform and high capacity loading of amino groups. Once the beads were deemed adequate for the linking of small molecules by the coupling of NHS-fluorescein followed by microscopy, chemical hydrolysis and fluorometry, the flavonoid naringenin was modified with 1,4-dibromobutane, followed by the attachment of aminopropyltriethoxysilane. After NMR structural confirmation, the resulting 7-(4-(3-(triethoxysilyl)propylamino)butoxy) naringenin was attached to the ceramic beads.

**Conclusion:**

Our results demonstrate that ceramic and glass beads provide convenient solid supports for the efficient and facile coupling of small molecules. We succeeded in generating naringenin-coupled ceramic and glass beads. We also developed a convenient series of steps that can be applied for the solid-support coupling of other related flavonoids. The availability of solid-support coupled naringenin opens up new opportunities for the identification of flavonoid-binding proteins.

## Background

Natural products are small molecules synthesized by bacteria, algae, fungi and plants, as part of their biotic and abiotic interactions with the environment. They provide a valuable source of pharmaceuticals, with 45% of the most popular drugs derived from natural products [1]. However, the identification of molecular targets for natural compounds with pharmacological activity remains a significant bottleneck in the process of drug validation. Plants provide a formidable source of natural products (phytochemicals) with pharmacological activity [2]. Over 100,000 phytochemicals have already been identified from the small fraction of the plant kingdom that has so far been surveyed [3]. These phytochemicals can be classified into large groups that include the alkaloids, the terpenoids, and the phenylpropanoids.

Flavonoids, derived from phenylpropanoids, are widely distributed throughout the plant kingdom and are abundantly present in many fruits and leaves [4]. They are characterized by the presence of two benzene rings linked by a pyrane or pyrone ring (ring C). Based on the position and modifications of the A, B and C rings, the 4,000+ flavonoids so far known can be classified into several sub-classes including the flavonols, the flavones, the isoflavones and the red/purple anthocyanin pigments. Flavonoids are potent antioxidants [5] and have important activities as dietary anti-carcinogens and anti-inflammatory compounds [6-10]. Flavonoids are also significant to plants, serving as signal molecules in various developmental processes [11]. Several studies have investigated the effect of flavonoids on the activity of various enzymes. For example, flavonoids possess protein kinase [12-15] and P-glycoprotein [16] inhibitory activities. However, with a few exceptions, *in vivo *animal and plant cellular targets for flavonoids are largely unknown.

Few methods are currently available for identifying proteins that bind to small molecules of plant origin, for example flavonoids. The development of high-throughput methods for the identification of flavonoid-binding proteins could significantly advance our understanding of the mechanisms by which flavonoids modulate plant hormone transport [17], contribute to plant male fertility [18], serve as allelochemicals [19] and facilitate the identification of missing metabolic enzymes in the flavonoid biosynthetic pathway. One promising approach is the generation of flavonoid derivatives that contain a benzophenone chromophore for use as a photoaffinity reagent [20]. While benzophenone-modified flavonoids can potentially permit the identification of flavonoid-binding proteins, they are inadequate for the isolation of significant quantities of proteins, necessary for their mass spectrometry identification. The isolation of flavonoid-binding proteins would be simplified by the availability of flavonoids covalently-linked to solid supports that would permit the affinity-purification of flavonoid-binding proteins from complex protein mixtures or protein libraries. Few methods are currently available that explore the possibility of linking select flavonoids to solid supports.

Here, we describe the efficient coupling of the flavanone naringenin, a central intermediate in the flavonoid biosynthetic pathway [21], to small ceramic and glass beads. The method developed can be easily adapted for the coupling of other related flavonoids to beads. The naringenin-coupled beads provide a powerful chemical genetic tool to probe biological systems.

## Results and discussion

### Ceramic and glass beads provide high-capacity solid supports

**Figure 1:**
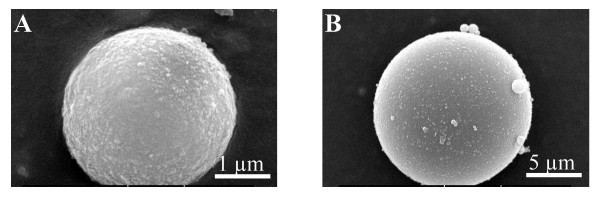
**Scanning electron micrograph of ceramic and glass beads. **Scanning electron micrograph of a representative (A) ceramic bead and (B) glass bead.

Many beaded solid supports are available, primarily developed towards combinatorial chemistry efforts [22]. The possibility to use small ceramic and hollow glass beads for the conjugation of small molecules was investigated here, because of their low cost, reduced size and compatibility with most organic and inorganic solvents. Scanning electron microscopy (SEM) experiments (Fig. 1) showed that the ceramic beads have an average particle volume of 5 μm^3 ^and a diameter of approximately 2 μm, about 5 times smaller than the glass beads (Table 1). These dimensions make them much smaller than the usual solid supports utilized in solid phase synthesis. The SEM analyses (Fig. 1A, B) also confirmed that both the ceramic and glass beads are not porous. This is an important property to consider given the ultimate goal to use the beads to identify proteins that bind to the small molecule, which requires a minimal hindrance of the small molecule by the bead matrix.

**Table 1:** Comparison of amino loading on the ceramic beads and glass beads

	Ceramic beads	Glass beads
Fluorescein loading (μmol/g)	0.34	0.03
Diameter of bead (μm)	2	10
Density of bead (g/cc)	2.5	1.1
Numbers of -NH_2 _group loading per bead	4 × 10^6^	1 × 10^7^
Surface area on bead for each – NH_2 _group (Å^2^)	250	3000

**Figure 2:**
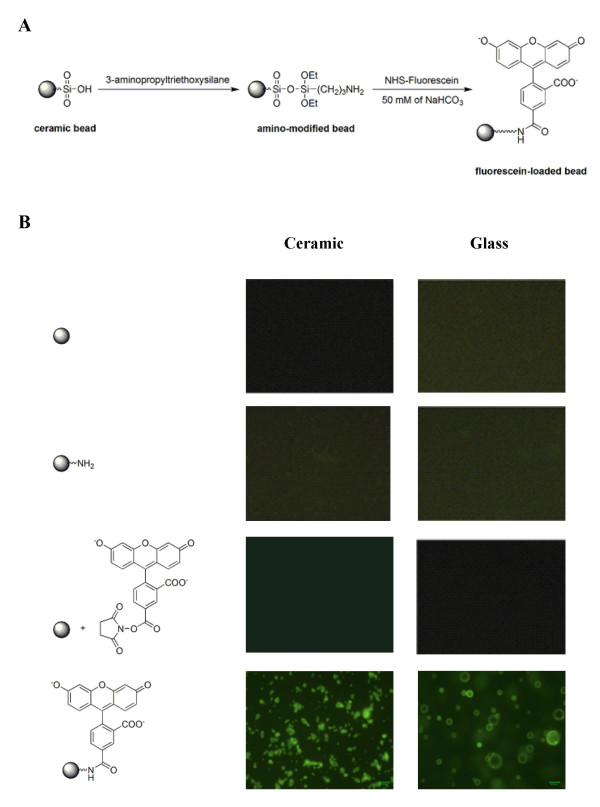
**Modification and fluorescein-loading of ceramic beads. **(A) Reaction scheme for the loading of amino groups on the ceramic and glass beads and the subsequent reaction with NHS-fluorescein. (B) Fluorescence micrographs of ceramic beads and glass beads.

The presence of Si-OH groups on the surface of the beads allows the rapid conjugation of bifunctional organosilanes [23,24]. To investigate the capacity of the loading of functional groups onto the surface of the ceramic and glass beads, silanisation of the beads was performed using 3-aminopropyltriethoxysilane (Fig. 2A). After washing and drying completely, the amino-modified beads were stored at 4°C until use.

In order to determine the effective loading of amino groups on the beads, both naïve (unmodified) and amino-modified glass and ceramic beads were incubated with NHS-fluorescein in the dark at 4°C for 24 hrs. After completion of the reaction, the beads were washed with water and dried. Naïve glass or ceramic beads displayed no fluorescence, nor did they fluoresce after incubation with NHS-fluorescein, while amino-modified glass and ceramic beads fluoresced after reaction with fluorescein (Fig. 2B). To quantitatively analyze the loading of amino groups on the surface of the beads, fluorescein was cleaved off by treatment with 1N hydrochloric acid. After the solution was neutralized to pH 9, the released fluorescence was quantified by fluorometry. For the ceramic beads, the loading of fluorescein, which represents the number of amino groups on the beads available for reaction, was established to be 0.34 μmol/g, which is the minimum possible loading of amino groups on the beads. Since the density of ceramic beads is 2.5 g/cc and the average diameter of bead is 2 μm, it was estimated that there is one amino group every approximately 250 Å^2 ^of surface area, with a loading capacity of around 4 × 10^6 ^amino groups per ceramic bead (Table 1). Similar calculations estimated the capacity of the glass beads at 1 × 10^7 ^amino-groups per bead. To further investigate the distribution of the amino loading on the ceramic and glass beads, FACS flow cytometry was utilized. After the reaction with NHS-fluorescein, over 90% of the ceramic and glass beads displayed fluorescence (data not shown). Thus, not only were the beads efficiently loaded with amino groups, but this loading was also uniformly distributed among the beads. Together, these results indicate that both the ceramic and the glass beads met the desired requirements for serving as solid supports for the coupling of flavonoids, and the amino-modified beads have further potential for solid phase synthesis.

### Modification and coupling of naringenin to the solid support

Initially, it was envisioned that the amino-loaded ceramic beads could be used for the direct coupling of naringenin, in the presence of a convenient linker such as dimethyl suberimidate (DMS). This approach, however, resulted to be impractical because technical limitations prevented the quantification of the amount of naringenin coupled to the beads, and it was feared that non-reacted amino groups on the beads or imidoester groups on the DMS linker would react with components of the biological system (*e.g*., proteins) that were intended to be probed.

**Figure 3:**
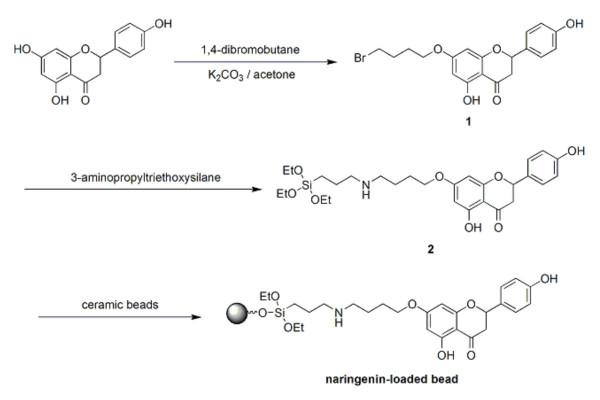
**Modification and loading of naringenin to ceramic and glass beads. **Reaction scheme for the loading of naringenin onto the ceramic and glass beads. Compound (1) corresponds to 7-(4-bromo-n-butoxy) naringenin and compound (2) corresponds to 7-(4-(3-(triethoxysilyl)propylamino)butoxy) naringenin.

Therefore, a different strategy was utilized, where the linker was first linked to the organosilane group on one end and naringenin on the other (Fig. 3), followed by the coupling of the entire moiety to the beads. One equivalent of 1,4-dibromobutane was first reacted with naringenin (Fig. 3), resulting in the formation of 7-(4-bromo-n-butoxy)naringenin (**1**) with little amount of 7,4'-di(4-bromo-n-butoxy) naringenin as a by-product [25]. After separation by chromatography on a silica column and structural elucidation by NMR (see Material and methods), the 7-(4-bromo-n-butoxy)naringenin was reacted with aminopropyltriethoxysilane, to give compound (**2**). The compound was purified by chromatography on silica gel and confirmed to be 7-(4-(3-(triethoxysilyl)propylamino)butoxy) naringenin by NMR. Once the identity of compound (**2**)was confirmed, its coupling to sodium hydroxide-treated ceramic beads in 95% methanol was performed overnight. Separation of the beads from non-conjugated compound (**2**)was accomplished by filtration and washing.

**Figure 4:**
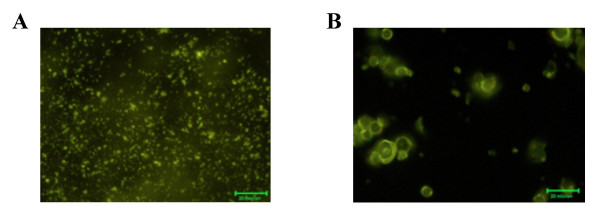
**Fluorescence micrographs of compound (2)-loaded beads. **Compound (2)-loaded (A) ceramic beads and (B) glass beads treated with NHS-fluorescein.

To ensure that compound (**2**)(Fig. 3) was coupled to the beads, advantage was taken from the reactivity of NHS-fluorescein with the -NH- group present in 7-(4-(3-(triethoxysilyl)propylamino)butoxy) naringenin (Fig. 3). The fluorescence detected is indicative of the coupling of the 7-(4-(3-(triethoxysilyl)propylamino)butoxy) naringenin to the ceramic and glass beads (Fig. 4).

**Figure 5:**
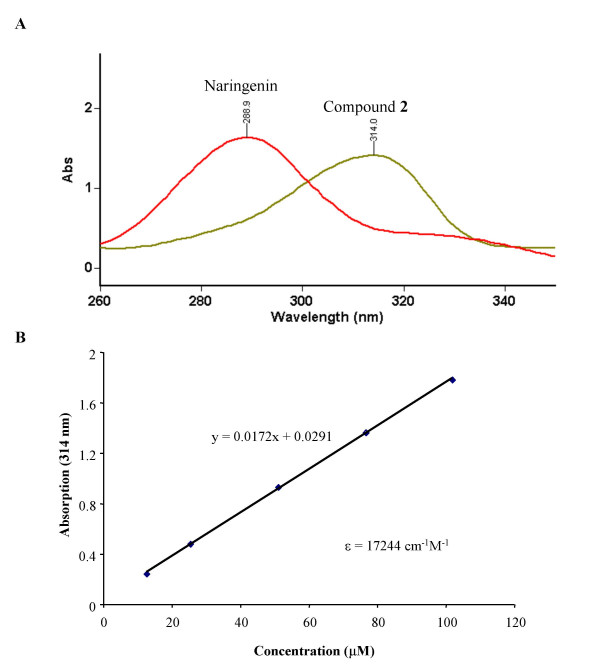
**Absorption properties of compound (2) **(A) Absorption spectra of naringenin and compound (2). (B) Calculation of the molar extinction coefficient (ε) for compound (2) at 314 nm.

To further quantify the amount of 7-(4-(3-(triethoxysilyl)propylamino)butoxy) naringenin coupled to the beads, the molar extinction coefficient (ε) of compound (**2**)was first calculated at 314 nm, corresponding to one of the absorption peaks of this compound (Fig. 5). The ε value of 17,244 cm^-1^M^-1 ^was used to calculate the amount of compound (**2**)that remained in solution after a coupling reaction was carried with a slight molar excess of 7-(4-(3-(triethoxysilyl)propylamino)butoxy) naringenin (assuming a bead capacity of 0.34 μmol/g). The loading of compound (**2**)was established to be 0.465 μmol per gram of beads, corresponding to approximately 5.5 × 10^6 ^molecules of 7-(4-(3-(triethoxysilyl)propylamino)butoxy) naringenin per ceramic bead. Using a similar approach, the coupling of 7-(4-(3-(triethoxysilyl)propylamino)butoxy) naringenin to the glass beads was established to be 0.00016 μmol/g, corresponding to 5.3 × 10^4 ^molecules per glass bead.

## Conclusion

The development of a simple and reliable method to link flavonoids to solid supports, in this particular case, small glass and ceramic beads, is described here. Interestingly, ceramic beads provided a much more robust solid support for coupling naringenin than the glass beads. These naringenin-coupled ceramic beads provide a convenient tool for the identification and isolation of naringenin-binding proteins. Although these studies describe only the coupling of naringenin to the beads through the 7-OH group in ring A, the possibility to selectively protect the various -OH groups offers opportunities for presenting the different groups in naringenin for recognition by proteins.

## Methods

### Microscopy

Scanning electron micrographs were obtained on a Philips XL Series Scanning Electron Microscope (SEM). Fluorescence micrographs were obtained on a Nikon Eclipse E600 (Nikon, Japan) microscope equipped with a OSRAM HBO Mercury Short Arc Lamp (λ_ex _485 nm, λ_em _515 nm, Mercury 100 W, CHIU Technical Corporation). Fluorescence was measured in a FLEX Station™ Instrument (λ_ex _491 or 496 nm, λ_em _518 or 519 nm, Molecular Devices) with the SOFTmax^@^PRO program.

### ^1^H, ^13^C NMR and UV spectra

^1^H and ^13^C NMR spectra were recorded on a Bruker NMR spectrometer (DRX-250 and DRX-500) in acetone-d6 (Aldrich). UV absorption spectra were performed on a Cary 50 Bio UV-Visible spectrophotometer. Data was analyzed by the Varian Cary WinUV Scan application.

### Chromatography methods

Column chromatography was performed on silica gel (Aldrich, 200–400 mesh) using the indicated solvents. TLC analyses were carried out using silica gel plates (Aldrich).

### Amino-modification of ceramic beads

Five grams of ceramic beads (3M™ Zeeospheres™ Ceramic Microspheres) or glass beads (Aldrich) were shaken in 20 mL of 10% sodium hydroxide solution overnight, and subsequently washed with water (40 mL), 1% hydrochloric acid (40 mL), water (40 mL) and methanol (40 mL), three times each. Then, the ceramic beads were shaken in 30 mL of 3% aminopropyltriethoxysilane solution made in 95% methanol at 4°C overnight, followed by washing with methanol (40 mL) and water (40 mL), three times each. After air-drying, the amino-modified ceramic beads were stored at 4°C.

### Fluorescein-coupled ceramic beads

Amino-modified ceramic beads (0.3 g) were added to 1 mL of 50 mM sodium bicarbonate buffer (pH ~8.5). In a dark room, 0.2 mg of NHS-fluorescein (Pierce Biotechnology, Inc.) were dissolved in 200 μL of DMSO, and then the NHS-fluorescein solution was added to the sodium bicarbonate suspension of beads and mixed well. The suspension was protected from light and shaken at 4°C for 24 hrs. After the liquid was removed by centrifugation and washed extensively with water, the fluorescein-bound ceramic beads were dried and stored at -20°C for future use. To compare with the control, bare (not amino group loaded) ceramic beads were treated under the same conditions. To test the fluorescence of bare and modified beads, all beads were separately shaken in a mixture of 200 μL of DMSO and 1 mL of 50 mM sodium bicarbonate buffer and dried.

### Hydrolysis of fluorescein from ceramic beads

Fifty mg of fluorescein-loaded ceramic beads were shaken in 1 mL of 1 N hydrochloric acid solution at 4°C for 4 days in the dark. After filtration through a 0.45 μm cellulose filter and washed with water, the filtrate was neutralized with 50 mM sodium bicarbonate buffer. To compare with the control, all bare beads incubated with or without fluorescein were treated under the same conditions. Fluorescence was measured on 200 μL of serial cleavage solution for each kind of beads in a 96 well cell microplate from both the top and bottom faces. A pure NHS-fluorescein solution was prepared as a calibration standard to calculate the absolute amount of fluorescein released form the beads.

### Preparation of 7-(4-bromo-n-butoxy)naringenin (1)

Naringenin (136 mg, 0.5 mmol, 1.0 eq) was dissolved in acetone (5 mL), then potassium carbonate (69 mg, 0.5 mmol, 1.0 eq) was added and heated to reflux for 30 minutes. To this suspension, 1,4-dibromobutane (107 mg, 0.5 mmol, 1.0 eq) was added and stirred at reflux for 8 hours. After cooling to room temperature, the mixture was concentrated on a rotary evaporator to become a thick slurry. The slurry was re-dissolved in ethyl acetate (30 mL), washed with distilled water (three times with 10 mL) and brine (10 mL), then evaporated. Purification was performed by silica-gel column chromatography (3:1 hexanes/ethyl acetate) to give pure 7-(4-bromo-n-butoxy)naringenin as a white solid (132 mg, 65% yield): ^1^H NMR (acetone-d6, 250 MHz) δ 1.86–1.97 (m, 2H), 2.00–2.09 (m, 2H), 2.74 (dd, *J *= 17.0, 3.0 Hz, 1H), 3.19 (dd, *J *= 17.0, 12.8 Hz, 1H), 3.57 (t, *J *= 6.5 Hz, 2H), 4.11 (t, *J *= 6.3 Hz, 2H), 5.46 (d, *J *= 12.5 Hz, 1H), 6.03 (s, 1H), 6.04 (s, 1H), 6.89 (d, *J *= 8.3 Hz, 2H), 7.38 (d, *J *= 8.3 Hz, 2H), 8.48 (s, 1H), 12.11 (s, 1H); ^13^C NMR (acetone-d6, 62.9 MHz) δ 28.34, 30.18, 34.27, 43.46, 68.38, 79.98, 94.94, 95.88, 103.72, 116.17, 128.99, 130.67, 158.68, 164.15, 164.95, 168.09, 197.53.

### Preparation of 7-(4-(3-(triethoxysilyl)propylamino)butoxy) naringenin (2)

7-(4-bromo-n-butoxy)naingenin (50 mg, 0.12 mmol, 1.0 eq) and pyridine (28 mg, 0.36 mmol, 3.0 eq) were dissolved in dry DCM (1 mL), 3-aminopropyltriethoxysilane (32 mg, 0.14 mmol, 1.2 eq) was then added and shaken for 48 hrs. After removal of DCM, the residue was purified by silica-gel column chromatography (2:1 hexanes/ethyl acetate) to give pure 7-(4-(3-(triethoxysilyl)propylamino)butoxy) naringenin as a pale yellow solid (15 mg, 22% yield): ^1^H NMR (acetone-d6, 500 MHz) δ 0.72 (t, *J *= 8.2 Hz, 2H), 1.16 (t, *J *= 6.7 Hz, 9H), 1.42 (m, 2H), 1.79 (m, 2H), 1.89 (m, 2H), 2.9 (dd, *J *= 16.8, 12.2 Hz, 1H), 3.20 (dd, *J *= 16.9, 3.0 Hz, 1H), 3.48 (m, 2H), 3.54 (m, 2H), 3.80 (q, *J *= 7.2 Hz, 6H), 4.00 (t, *J *= 6.3 Hz, 2H), 5.12 (dd, *J *= 12.2, 2.8 Hz, 1H), 5.77 (d, *J *= 2.3 Hz, 1H), 5.88 (d, *J *= 2.3 Hz, 1H), 6.88 (d, *J *= 8.6 Hz, 2H), 7.36 (d, *J *= 8.5 Hz, 2H), 8.58 (s, 1H). This is an easy, but not efficient procedure. We successfully completed this reaction independently twice, however, we failed in other opportunities to obtain the desired product for unexplained reasons.

### Coupling of naringenin to ceramic beads

Ceramic beads (30 mg) were first treated with 10% sodium hydroxide solution and washed with water, hydrochloric acid and methanol as above, then shaken with 7-(4-(3-(triethoxysilyl)propylamino)butoxy) naringenin (20 mg) in 95% methanol (1 mL) for 24 hrs. After filtration, the naringenin-coupled ceramic beads were washed three times each with methanol, water and methanol, then vacuum dried. The obtained beads were treated with NHS-fluorescein and detected by fluorescence microscope, as described above.

### Determination of naringenin loading on ceramic beads

7-(4-(3-(triethoxysilyl)propylamino)butoxy) naringenin (0.164 mg, 0.3 μmol) was shaken with ceramic beads (500 mg) in 3 mL of 95% methanol for 24 hrs. After filtration, 1 mL of the filtrate was measured by absorbance at 314 nm. 7-(4-(3-(triethoxysilyl)propylamino)butoxy) naringenin (compound **2**) was dissolved in 95% methanol (concentration from 128 μM to 12.8 μM) and measured as a calibration. Based on Beer's law, the molar extinction coefficient (ε) was determined to be 17244 cm^-1^M^-1^.

## Authors' contributions

YL carried out all the chemical experiments described, NGI helped with the fluorescence microscopy and fluorometry. EG conceived the project and participated in the design and coordination of the study.

**Table 2:** Comparison of naringenin loading on the ceramic and the glass beads

	Ceramic beads	Glass beads
Naringenin Loading (μmol/g)	0.465	0.00016
Numbers of naringenin loading on per bead	5.5 × 10^6^	5.3 × 10^4^
Surface area on bead for each naringenin molecule (Å^2^)	180	5.7 × 10^5^
